# Peer-instructed seminar attendance is associated with improved preparation, deeper learning and higher exam scores: a survey study

**DOI:** 10.1186/s12909-016-0715-0

**Published:** 2016-08-09

**Authors:** Rianne A. M. Bouwmeester, Renske A. M. de Kleijn, Harold V. M. van Rijen

**Affiliations:** 1 Department of Medical Physiology, Division Heart and Lungs, University Medical Center Utrecht, Yalelaan 50, 3584CM Utrecht, The Netherlands; 2 Center for Education and Training, University Medical Center Utrecht, Utrecht, The Netherlands; 3 Centre for Teaching and Learning, Utrecht University, Utrecht, The Netherlands

**Keywords:** Active learning, Student motives, Participation, Engagement, Small-group seminar learning

## Abstract

**Background:**

Active engagement in education improves learning outcomes. To enhance active participation in seminars, a student-centered course design was implemented and evaluated in terms of self-reported preparation, student motivation and exam scores.

We hypothesized that small group learning with intensive peer interaction, using buzz-groups followed by plenary discussion, would motivate students to prepare seminar assignments at home and to actively engage in the seminars. Active engagement involved discussion of the preparatory assignments until consensus was reached.

**Methods:**

In total seven seminars were scheduled in a 10-week physiology course of an undergraduate Biomedical Sciences program. After each seminar, students were asked to fill out their perceptions of preparation and quality of the seminar (deepening of knowledge and confidence in answers) on a five-point scale using electronic questionnaires. Student motives were first collected using open questions. In the final questionnaire students were asked to indicate on a five-point scale how each motive was perceived. Students overall explanations why they had learned from seminars were collected via open questions in the final questionnaire. One hundred and twenty-four students of the cohort from November 2012 to February 2013 (82.6 %) voluntarily participated. Students’ motives to prepare and attend seminars were analyzed by inspection of descriptive statistics. Linear regression analysis was conducted to relate student preparation to the quality of seminars, seminar attendance to exam scores, and exam scores to the quality of seminars. Answers to open questions were deductively clustered.

**Results:**

Studying the material, training for exams and comparing answers with peers motivated students to prepare the seminars. Students were motivated to participate actively because they wanted to keep track of correct answers themselves, to better understand the content and to be able to present their findings in plenary discussions.

Perceived preparation of peers was positively associated with the perceived quality of seminars. Also, seminar attendance was positively associated with exam scores. Students’ overall explanations suggest that discussing with peers and applying knowledge in pathophysiology cases underlies this association.

**Conclusion:**

Discussion with well-prepared peers during seminars improves student perceptions of deeper learning and peer-instructed seminar attendance was associated with higher exam scores.

**Electronic supplementary material:**

The online version of this article (doi:10.1186/s12909-016-0715-0) contains supplementary material, which is available to authorized users.

## Background

Active participation in educational activities is essential for learning [1–3] since active participation improves students’ level of understanding, the ability to process material, and the retention of knowledge [4–6]. As a consequence exam scores of students participating in active learning environments are often higher than those of students attending traditional lectures [6]. However, not all students engage in learning activities and are, therefore, usually identified as passive recipients of information [7]. Lack of preparation, most likely caused by low levels of motivation is a probable cause for students not to participate actively in learning activities [8]. As a consequence, these students are assumed to learn less than students who participate actively in learning activities.

The importance of active learning has also been acknowledged in (bio-) medical education [3]. Medical education settings have, therefore, changed the traditionally passive faculty-centered environments into active student-centered learning environments [9, 10]. Next to small group learning or problem-based learning, seminar learning is an important form of an activating student-centered learning environment [11, 12].

During seminar learning, groups of (25–30) students discuss questions and issues under supervision of a content expert [13]. An important aim of seminar learning is to enhance peer-instruction, which involves students to explain topics to one another using their own words. These explanations should then be very specific and concrete [14, 15]. Peer-instruction is known to promote long-term memory [16, 17] and provides students with insight into their level of understanding and their performance. In addition, teachers can identify student’s needs, determine how students assimilate information and indicate future learning directions [7].

Spruijt and co-workers have identified three factors for successful seminar learning [18, 19]. First, the preparation material as well as the seminar assignments should be manageable and of good quality according to both students and teachers. Second, seminars should be well structured and clearly connected to other educational methods; a characteristic also known as constructive alignment [20] since students are likely to adopt a surface approach to learning when constructive alignment is lacking [21]. The third requirement for efficient seminar learning is a course schedule that allows sufficient time to prepare seminar assignments. Students considered preparation to have a major impact on their learning, because it enhanced the value of participating in the seminar [18].

Interestingly some discrepancies between educational theory and practice have been described [22, 23]. For example, Jaarsma and co-workers observed that interactions within seminars were predominantly between the teacher and the students while few student–student interactions occurred meaning less opportunity for peer-instruction [24].

Similar to the descriptions of Jaarsma et al., seminars in the current study were aimed at deep learning by peer-discussion. Even though these seminars were based on pre-assigned seminar assignments instead of literature readings [13], teachers observed that only a limited number of students fully prepared the seminar assignments and few student-student interactions occurred.

In this study we investigated if a student-centered design stimulated students to prepare and participate actively in seminar learning, leading to improved academic outcome. We made the seminar groups seem smaller [25] by introducing buzz groups [26] followed by a plenary peer-driven discussion. We hypothesized that the combination of smaller groups and a highly aligned educational design would positively influence study behavior, such that students would prepare the assignments, more student-student interactions would occur, and learning outcome would improve. In addition, we aimed to unravel the underlying mechanism, by investigating what stimulated and reduced students’ motivation to prepare for and participate actively in seminars.

## Methods

### Educational setting

This survey study was conducted in 2013 in a second-year undergraduate 10-week physiology course, named ‘Organ Systems’, of the undergraduate program Biomedical Sciences at Utrecht University in the Netherlands. The course load was 20 h per week (i.e. 50 % of student time) and is composed of four parts. First, three physiology-oriented parts focus sequentially on the Respiratory, Circulatory, and Urinary organ systems, which were each finalized with a multiple-choice (MCQ) exam, followed by an integrative part about the pathophysiology of heart failure. All parts took two weeks, except for the Urinary system, which took three weeks. The final week of the course was spent on self-directed preparation for the final exam, consisting of open-ended essay questions.

In the three physiology-oriented parts, each week, six of the 20 h were spent as contact sessions between students and teachers, including one seminar per week. The seminars were not obligatory, but attendance was strongly encouraged by the coordinator of the course. Each seminar was scheduled for 2 h on the day after a lecture on the same topic. The remaining 14 h were available for preparation for the lectures, seminars, summative MCQ and essay exams.

### Seminars

To enhance student preparation and active participation, the format of seminars was redesigned. First, students could individually prepare the six seminar assignments as homework. The answers on assignments were than discussed in buzz groups of ideally five students [27] in the first part of the seminar. Students were thereby encouraged to compare and discuss their answers until consensus was reached. During the second part of the seminar, each buzz group was assigned to present their answer to one of the six seminar assignments to the whole group of 30 students. Their answer was reviewed and supplemented by other buzz groups in a plenary discussion. The seminar teacher moderated the classroom discussions and kept track of the answers to ensure they were correct and complete.

Finally, students could recapitulate their seminar answers supplemented with peer comments by writing a summary of their presented assignment on a so-called *wiki*, which was part of the online learning environment. Every seminar group had its own wiki, so in total there were five wikis. Together, the answers of all seminar assignments represent a clear overview of the main topics taught during the course.

### Participants

Yearly, 150 students enroll in the physiology course Organ systems. To stimulate peer-instruction in seminars, the 150 students were divided into five groups of 30 students and every group was further divided into six buzz groups of five students [25, 27]. The composition of these buzz groups was deliberately maintained over the duration of the course, to stimulate students to improve their performance and participation in the seminars.

One hundred and twenty-four students of the cohort from November 2012 to February 2013 (82.6 %) participated in this study and all seminar groups were represented. Participation was voluntarily and students who filled out six or seven questionnaires were given a €10 gift certificate as compensation for their time.

A content expert guided each seminar. In total three biologists taught the respiratory and cardiovascular seminars and three veterinarians were renal experts.

### Alignment

Seminar assignments were redesigned and constructively aligned to the cognitive learning and thinking activities of the summative essay exam, which reflect the learning goals of the course, using a coding scheme described by Overman et al. [28]. Learning activities stimulating students to *relate, structure, analyze, apply* and *concretize* were aimed for. Two educational researchers inspected the overlap between exam items, seminar assignments and learning goals. Teachers approved the overlap.

### Procedure

To determine students’ self-reported study behavior, participants were invited to fill out an online questionnaire consisting of Likert-type items and open questions after every seminar (Additional file 1). For the final questionnaire, based on answers given in all previous questionnaires, the open questions were transformed into statements to be rated on five-point scales. After linking the questionnaire data to students’ exam scores and previous test scores, data were anonymized for further analyses. On average 102 students (82.3 %) filled out each online questionnaire. Twenty-six students filled out all seven questionnaires.

#### Data collection

First, with respect to student preparation, after each of the seven seminars students were asked if they were able to complete the assignments individually (yes or no) and how many hours they spent on preparing the seminar assignments (open). Next, they were asked to indicate on a five-point scale how well they perceived their peers to have prepared the seminar assignments (the endpoints of this scale were anchored as 1 = very poorly and 5 = very good). Additionally, students were asked six times to indicate what increased and/or decreased their motivation to prepare the seminar assignments. In the final questionnaire, students were asked to indicate on five-point scales how each motivating and demotivating item was perceived (the endpoints of these scales were anchored as 1 = little contribution/little reduction and 5 = large contribution/large reduction). An overview of the items in the online questionnaires is shown in Additional file 1.

Second, with respect to the actual seminars, student attendance was measured by headcount. Also, student motivations and demotivations to participate actively in seminars were collected similar to motivations for preparation (using anchored scales with 1 = little contribution/little reduction and 5 = large contribution/large reduction). Concerning demotivation, the answers provided by the subgroup of students who chose not to attend the last three seminars, were analyzed separately as well. In addition, student perceptions of the quality of the seminars were measured on a five-point scale in the online questionnaires with the following two questions: ‘*To what extent did the seminar deepen your knowledge of topics discussed during the lecture?*’ and ‘*To what extent are you confident that you and your peers found the correct answer?*’ Both scales were anchored with 1 = very little and 5 = very much.

Third, the summative exam scores were collected of participating students and as a proxy measure for previous academic achievement, students’ course grades of six previous but related courses in their first year of Biomedical Science education were collected and averaged.

### Student and teacher perceptions of the quality of the seminars

Students were asked to describe how much they had learned from the seminars in general and to elaborate on their answer in the final online questionnaire. This question was answered by 91 students (73.4 %). Some students provided more than one explanation. Students’ explanations were deductively clustered after carefully reading all responses to this question. An education researcher checked if clustering was logical and if themes were comprehensive.

In addition to student evaluation, all six teachers were asked to fill out a short open-ended questionnaire to determine their experience of the new seminar format and their perceptions of students’ preparation and participation (The questionnaire is shown in Additional file 2). The questionnaire consisted of seven open-ended questions. Besides two general questions, teachers were asked how they experienced teaching the seminars and to describe which changes they had observed in student behavior and which expected behavior was missing. In addition, teachers were asked to determine advantages and disadvantages by comparing the new seminar format to the traditional seminars guided in previous cohorts. The response rate to this questionnaire was 100 %.

Teacher responses to the questionnaire were thoroughly discussed in the research team. In the discussion main advantages and disadvantages were identified and clustered.

### Statistical analyses

#### Descriptives

Self-reported preparation time and the five-point scale items (perceived preparation of peers, deepening of knowledge and confidence in correct answers) were analyzed by inspection of frequencies and descriptive statistics using SPSS 20.0 [IBM Corporation, New York, USA]. Motives to prepare and motives to participate actively were analyzed similarly. We determined the percentage of students that were explicitly positive, in other words who responded with a 4 or 5. When this proportion was larger than 50 % we interpreted the motives to be largely shared by students.

Four variables (i.e. preparation time, perceived preparation of peers, confidence in answers and deepening of knowledge) were measured in all seven questionnaires. In order to use this variable in the regression analyses, we had to transform these seven measurements into one score. Therefore, we calculated the average perception of each variable. However, the measurements of the fifth seminar were excluded, because the low participation rate induced teachers to use a different teaching strategy. Instead of the procedure described above, teachers were directly discussing the seminar assignments with all attendees.

#### Regression analyses

First, the association of seminar preparation with the perceived quality of the seminar sessions was investigated. Therefore, we used as dependent variables perceived ‘deepening of knowledge’ and ‘confident in answers’. Independent variables were self-reported preparation time and the perceived preparation of peers.

Second, the association of seminar attendance with performance on the summative exam was investigated. The summative exam score was the dependent variable and number of seminars attended was the independent variable. Additionally, in this model we controlled for previous academic achievement, as we would expect the students who are generally more motivated and more intelligent would attend more seminars and, therefore, score better on the exam in any situation.

Lastly, the association of students’ performance on the summative exam with the perceived quality of the seminar sessions was investigated. Therefore, exam scores were used as the dependent variable, and student perceptions of ‘deepening of knowledge’ and ‘confidence in answers’ were used as independent variables. Again, previous academic achievement was controlled for. *P*-values < 0.05 were considered statistically significant.

## Results

### Alignment

**Fig. 1:**
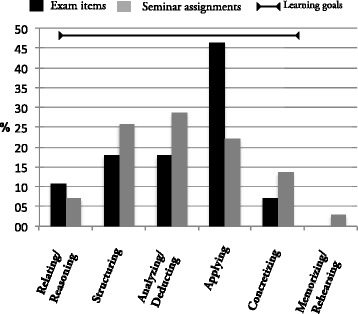
Alignment of exam items and seminar assignments to learning goals. All exam items (*black*) and most seminar assignments (*grey*) overlap with the intended learning goals indicated with ‘’. Y-axis represents percentages of items. Categories, in descending order of cognitive complexity, are indicated on the x-axis

As illustrated in Fig. 1, the 28 exam items (shown in black) covered all cognitive learning activities that were aimed for in the learning goals of the course. The overlap between the 140 seminar assignments (shown in grey) and the exam items was satisfactory to teachers.

### Preparation for seminars

As is shown in Table 1, self-reported preparation time was comparable for all sessions, on average students spent 1.59 to 1.91 h per seminar to prepare the assignments. Within this preparation time, 61–62 % of students completed the assignments of every first session of a new topic (session 1, 3 and 5). In contrast, 35–44 % completed this task for the final session of each topic (session 2, 4 and 7).

**Table 1:** Self-reported and perceived preparation

Session	Seminar	Self-reported participation (*N* (% total))	Completion (*N* (% participants))	Min (h)	Max (h)	Mean (SD) (h)	Perceived preparation peers	
							Mean (SD)	≥4 (%)
1	Resp 1	103 (83)	64 (62)	0	5	1.68 (1.00)	3.37 (1.02)	46.6
2	Resp 2	98 (79)	37 (38)	0	7	1.91 (1.09)	4.15 (0.88)	79.1
3	Circ 1	88 (68)	54 (61)	0	5	1.74 (0.99)	3.60 (0.92)	59.1
4	Circ 2	82 (66)	29 (35)	0	5	1.59 (1.07)	3.88 (0.90)	70.4
5	Uri 1	36 (29)	22 (61)	0	7	1.60 (1.43)	2.83 (1.13)	30.5
6	Uri 2	67 (54)	36 (54)	0	5	1.63 (0.95)	3.51 (0.89)	52.2
7	Uri 3	93 (75)	38 (44)	0	5	1.63 (1.35)	3.44 (1.08)	50.6

Self-reported participation and the completion of homework assignments was questioned using the multiple-choice options “Yes or No”. Preparation time was asked via a open question. The perceived preparation of peers was surveyed using an anchored scale ranging from 1 (very poorly) to 5 (very good). ≥ 4 (%) illustrates the percentage of students rating the Likert-items with a 4 or 5

More than 50 % of the students perceived the preparation of peers to be (very) good for most seminars. In the first session 47 % were perceive to be prepared good, whereas only 31 % was perceived prepared well for the fifth session. Students were most satisfied with the preparation of their peers during the second session, as 79 % of the students scored 4 or 5.

Comparison of self-reported preparation and perceived preparation of peers suggests that students were more satisfied with the preparation of their peers when a smaller number of students was able to complete the assignments at home (like sessions 2, 4 and 7). Conversely, students were less satisfied with the preparation of their peers, when a larger portion of students was able to complete the assignments on their own, as is the case in sessions 1, 3 and 5.

Table 2 shows that, with 78 % of the students scoring a 4 or 5, a main reason for preparing the seminar assignments was to study the material before the upcoming multiple-choice exam. Related to that, 73 % of the students claim to realize that seminar assignments would be a useful training for the end-of-course exams. In addition, 53 % of the students wanted to know whether they could complete the assignments on their own before discussing their answers with peers and 57 % wanted to compare their own answers to answers given by their peers. In general it seems that students’ motivation to prepare for seminars relate to their autonomy.

**Table 2:** Motivating and demotivating reasons to prepare for seminars

*To what extent do the following reasons improve your motivation to prepare the seminar assignments?*	Mean (SD)	≥4 (%)
I wanted to study the material before the upcoming multiple-choice exam	4.02 (0.93)	78.1
I realize that seminar assignments will be a useful training for the end-of-course exam	3.93 (0.99)	72.6
I wanted to compare my own answers to answers given by my peers	3.54 (1.09)	57.2
I wanted to know whether I could complete the assignments on my own before discussing them with my peers	3.51 (1.03)	52.8
I did not want to let my peers down	3.40 (1.00)	50.6
I find the content of this course interesting	3.34 (0.99)	49.5
I did not want to rely completely on the answers of my peers, because these might be incorrect or incomplete	3.25 (1.13)	47.3
I did not want to be identified as a free-rider	3.08 (1.25)	45.1
I knew that our teacher would not provide us the correct answers, therefore I wanted to find out what the correct answer was all by my self	2.63 (1.21)	27.5
The seminar assignments were challenging and therefore I liked to prepare them	2.70 (0.98)	18.7
I knew that our teacher would (informally) check whether I had prepared the assignments.	1.92 (1.09)	11.0
*To what extent do the following reasons decrease your motivation to prepare the seminar assignments?*	Mean (SD)	≥4 (%)
I prefer to spend time on other (learning) activities than to prepare the assignments	3.20 (1.23)	46.2
Seminar assignments are too difficult to prepare individually	2.97 (1.10)	33.0
Assignments will be repeated twice during the seminar (once in discussion with peers and again during plenary discussion)	2.59 (1.19)	28.6
I did not plan to go to the seminar	1.98 (1.28)	16.5
I knew that the correct answer would be place on the wiki’s	2.09 (1.12)	14.3
I planned to answers the assignment during the seminars	2.04 (1.11)	14.3
To me the assignment were not available in time	1.88 (1.23)	14.3
I knew the teacher would not provide us with answers or elucidations	2.06 (1.13)	12.2
I am not interested in the content of this course	2.12 (1.11)	11.0
My peer will immediately copy my answer	1.73 (1.11)	9.9
My peers will not take my answers seriously	1.36 (0.81)	3.3
I knew my peers would prepare the assignment, so there was no need to prepare them myself	1.63 (0.76)	2.2

Students could indicate to what extent every argument for improved or reduced motivation to prepare applied to them using an anchored scale ranging from 1 (little contribution/little reduction) to 5 (large contribution/ large reduction). ≥ 4 (%) illustrates the percentage of students rating the Likert-items with a 4 or 5

The majority of students did not score a 4 or 5 to one of the self-reported argument describing students reduced motivation. The most demotivating argument, to which 46 % of the students agreed, was that students preferred to spend time on (learning) activities other than preparing the assignments. In addition, 33 % of the students indicated that seminar assignments were too difficult to prepare individually, whereas 29 % of the students claimed that seminar assignments would be repeated twice during the seminar.

### Active participation during seminars

Headcount indicated that attendance of seminars decreased over time. Seventy-nine percent of the students attended both seminars concerning the Respiratory system, 71 % attended both seminars involving the Cardiovascular system, whereas 22 % (27 students) attended all three seminars involving the Urinary system. Likewise, the number of students attending neither of the seminars of a topic increased over time from 3 to 24 %.

The total number of answers to assignments shared via the wiki’s also reduced over time. On average 90 % of the answers were shared on wiki’s concerning the Respiratory system. Wiki’s regarding the Circulatory system contained 55 % of the answers, whereas only 37 % of the answers about the Urinary system were shared. However, the contribution to wiki’s varied largely between seminar groups. One group shared 98 % of the answers with their peers, while another group shared not more than 43 %.

### Perceived quality of the seminars

As shown in Table 3, students indicated that they were confident in finding the correct answers to the seminar assignments (all seminars were rated ≥ 4 by more than 50 % of the students). Similarly, students reported that all seminars had deepened their knowledge. The second seminar was most satisfying, based on the percentage of students scoring a 4 or 5, when considering the average scores students were most satisfied with the fifth seminar.

**Table 3:** Perceived quality of seminars

	Confidence in answers		Deepening of knowledge	
Seminar	Mean (SD)	≥4 (%)	Mean (SD)	≥4 (%)
Resp 1	4.03 (0.94)	77.7	3.94 (0.79)	79.7
Resp 2	3.94 (0.77)	79.2	4.06 (0.72)	88.5
Circ 1	3.42 (1.10)	52.3	3.90 (0.80)	76.2
Circ 2	3.89 (0.73)	72.8	3.86 (0.79)	75.3
Uri 1	4.17 (0.85)	77.8	4.03 (0.81)	75.0
Uri 2	3.85 (1.02)	68.7	3.79 (0.90)	70.1
Uri 3	3.69 (0.96)	57.2	3.41 (0.95)	49.5

The perceived confidence in finding the correct answer and the perception that seminars deepened student knowledge were surveyed using an anchored scale ranging from 1 (very little) to 5 (very much). ≥ 4 (%) illustrates the percentage of students rating the Likert-items with a 4 or 5

Table 4 shows students main arguments explaining their increased motivation to participate actively in seminars. The most important argument, according to 79 % of the students, was that they wanted to keep track of the correct answers themselves, therefore not being too dependent on the wikis. Additionally, 69 % of the students wanted to ‘better understand the content by discussing with their peers’ and 68 % of the students wanted to be able to present the correct answer during plenary discussions. In general, no elements were found that were highly demotivating for active participation during the seminars, although the most demotivating factor was the repetition of the assignments during the actual seminar, according to 21 % of all student.

**Table 4:** Motivating and demotivating reasons to actively participate in seminars

*To what extent do the following reasons improve your motivation to participate actively in seminars?*			Mean (SD)	≥4 (%)
I wanted to write down the correct answers myself, so I don’t have to rely on the wiki’s			3.99 (1.06)	79.2
I want to better understand the content by discussing with my peers			3.77 (1.02)	69.3
I want my subgroup to be able to present the correct answer during the plenary discussion			3.74 (0.92)	68.1
I wanted to be able to criticize the correctness and completeness of answers given by other subgroups			2.96 (1.02)	31.9
I knew the teacher would not provided us the correct answer, therefore I wanted to find out the correct answers myself			2.91 (1.12)	33.0
It was easier to ask the teacher for help, when the assignments were discussed with my peers			2.84 (1.15)	33.0
I expected that active participation would lead to goodwill of our teacher to provide us with additional clues			2.62 (1.16)	23.1
I wanted to find out which assignments should be discussed in the Meet The Expert session			1.68 (0.91)	4.4
*To what extent do the following reasons decrease your motivation to participate actively in seminars?*	Mean (SD)	≥4 (%)	Mean (SD) students not attending	≥4 (%)
I did not want to explain everything to my (unprepared) peers	1.91 (1.14)	12.1	2.36 (1.29)	27.3
Repetition of the assignments during the seminar; First in peer-discussion followed by plenary discussion	2.43 (1.16)	20.9	2.45 (0.93)	9.1
I knew that the correct answer would be placed on the wiki	1.98 (1.03)	7.7	2.18 (0.98)	9.1
I knew the teacher would not provide us with the correct answer	1.95 (1.04)	6,6	2.36 (1.21)	9.1
I am not interested in the content of this course	2.16 (1.07)	9.9	1.91 (0.83)	0
I knew my peers would prepare the assignments	1.90 (0.90)	5.5	1.73 (0.79)	0

Students could indicate to what extent every argument for active participation applied to them using an anchored scale ranging from 1 (little contribution/little reduction) to 5 (large contribution/large reduction. ≥ 4 (%) illustrates the percentage of students rating the Likert-items with a 4 or 5

From the subgroup of students, who choose not to attend the last three seminars, 9 % identified that repetition of assignment was demotivating. However, not wanting to explain everything to (unprepared) peers was rated a 4 or 5 by 27 % of this subgroup of students.

### Relation between students’ preparation and the quality of the seminar

To explore if preparation predicts perceived quality of seminars, regression analyses was performed. As shown in Table 5, model 1, perceived preparation of peers (B = 0.18; *p* = 0.02) could explain why students experienced that attending seminars deepened their knowledge (*R*^*2*^ = 0.09, *ΔR*^*2*^ = 0.08) and could also explain why students have confidence in having correct answers (B = 0.40; *p* < 0.01, *R*^*2*^ = 0.21, *ΔR*^*2*^ *=* 0.20) (Model 2).

**Table 5:** Regression analyses correlating variables of preparation with (1) deepening of knowledge and (2) confidence in correct answers

	1. Deepening of knowledge				2. Confidence in correct answers			
Variable	B	*p*	β	95 % CI	B	*p*	β	95 % CI
Constant	2.94	.00		[2.34, 3.54]	2.16	.00		[1.50, 2.81]
Preparation time	.15	.06	.18	[−.01, .31]	.11	.21	.11	[−.06, .28]
Perceived preparation peers	.18	.02	.24	[.03, .33]	.40	.00	.44	[.24, .57]
*R* ^*2*^	.09				.21			
*ΔR* ^*2*^	.08				.20			

Perceived preparation of peers relates to deepening of knowledge and confidence in answers

(*B* regression coefficient, *p* significance value, *β* standardized coefficient, *95 % CI* 95 % confidence interval, *R* ^*2*^ variance explained, *ΔR* ^*2*^ adjusted variance explained)

### Relation between seminar attendance and exam scores

Table 6 indicates that the number of seminars attended is positively associated with end-of-course exam scores (B = 0.34; *p* < 0.01, *R*^*2*^ = 0.20, *ΔR*^*2*^ = 0.19), even when corrected for previous achievement (B = 0.15; *p* = 0.02; *R*^*2*^ = 0.48, *ΔR*^*2*^ = 0.47).

**Table 6:** Regression analyses correlating exam scores with number of attended seminars

	Model 1					Model 2		
Variable	B	*p*	β	95 % CI	B	*p*	β	95 % CI
Constant	4.41	.00		[3.75, 5.07]	−1.98	.03		[−3.76, −.20]
*N* (seminars)	.34	.00	.45	[.22, .47]	.15	.02	.19	[.02, .27]
Previous achievements					1.10	.00	.60	[.81, 1.39]
*R* ^*2*^	.20				0.48			
*ΔR* ^*2*^	.19				0.47			

Number of seminars attended relates to (corrected) exam score

(*B* regression coefficient, *p* significance value, *β* standardized coefficient, *95 % CI* 95 % confidence interval, *R* ^*2*^ variance explained, *ΔR* ^*2*^ adjusted variance explained)

To further define the underlying mechanism, a model was tested in which ‘deepening of knowledge’ and ‘confidence in answers’ were used to predict exam scores (Table 7). However, these results were not significant, suggesting that other variables may explain the higher grades when students attend more seminars.

**Table 7:** Regression analyses correlating exam scores with variables of participation

	Model 1				Model 2			
Variable	B	*p*	β	95 % CI	B	*p*	β	95 % CI
Constant	6.84	.00		[4.93, 8.75]	−2.06	.12		[−4.67, .56]
Deepening knowledge	.03	.93	.01	[−.52, .58]	−.03	.90	−.01	[−.49, .43]
Confidence answers	−.17	.47	−.09	[−.64, .30]	.11	.63	.05	[−.33, .54]
Previous achievements					1.19	.00	.70	[.92, 1.46]
*R* ^*2*^	.01				.48			
*ΔR* ^*2*^	−.01				.46			

Deepening of knowledge and confidence in answers does not relate to exam scores

(*B* regression coefficient, *p* significance value, *β* standardized coefficient, *95 % CI* 95 % confidence interval, *R* ^*2*^ variance explained, *ΔR* ^*2*^ adjusted variance explained)

Lastly, students were asked to describe how much they had learned from the seminars in general. Table 8 shows that approximately 37 % of the students (*N* = 34) explained they had learned from the seminars because they could compare and discuss their answers with answers given by peers. Also 21 % report that seminars stimulated them to prepare and study the material before class. Additionally, 24 % of the students elucidate they were stimulated to apply their knowledge in pathophysiology cases.

**Table 8:** Students’ explanations for learning from seminars

I learned from the seminars, because …	N	%
I was able to compare and discuss my answers to answers given by my peers	34	37.4
I was stimulated to apply knowledge in pathophysiology cases	22	24.2
Seminars stimulate me to prepare and study the material before class	19	20.9
I was encouraged to actively process the material	12	13.2
Content was rehearsed/I was able to practice	11	12.1
They prepared me for the exam	10	11.0
Subjects are discussed more elaborately compared to the lectures	6	6.6

Clustering of students’ explanations why attending seminars were perceived useful. *N* = the number of students addressing each argument. Total number of respondents was 91. Some students provided multiple explanations. % represents the percentage of students that gave this explanation

### Teachers’ perception

Teachers described the new design of seminars as an improved format, which stimulated students to prepare better. They all noticed that students participated more actively during group discussions, compared to students in previous cohorts. Still, teachers were concerned with the decreasing attendance rates.

**Fig. 2:**
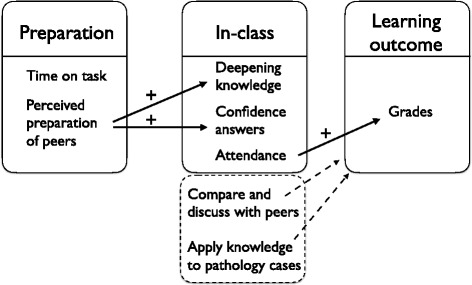
Model to illustrate the mechanism underlying student preparation, seminar attendance, and exam scores. Black arrows represent significant predictions (also indicated with ‘+’). Items in the dotted box represent factors that might have caused the improved learning outcomes according to students

Figure 2 illustrates how student preparation relates to the quality of seminars and that seminar attendance relates to exam scores.

## Discussion

The main findings of this study were that 1) seminar attendance is positively associated with exam scores, even when corrected for previous academic achievements and 2) perceived preparation of peers is associated with the perceived quality of seminars.

### Preparation and peer-discussion

Students’ preparation was relatively constant during this course, approximately 1.5 to 2 h per seminar, whether or not they were able to complete their homework. This is an interesting finding, since a large part of the obtained learning effect might be determined by students’ motivation to prepare [8]. The students participating in the present study claimed to be more motivated to prepare for the seminars because their performance in seminars could influence their autonomy. It seems that students use the seminar assignments to measure their own performance and compare this to a defined goal. This goal might be the level of understanding achieved by their peers, as students wanted to compare and discuss seminar answers with peers. Another goal might be the expected level of understanding required for the summative exam, as students used seminar assignments as training for the summative MCQ and end-of-course exams. These arguments correspond to the first of three scales of motivation identified by Aalbers, known as the ‘urge to learn’-scale [8]. Alternatively, the argument best explaining our students’ reduced motivation to prepare fitted nicely with Aalbers’ second category, i.e. students ‘lacked inner drive’ as they preferred to spend their time differently. Aalbers’ third category, expected difficulties, would correspond to students claiming that the assignments were too difficult. This third argument had a large contribution to 33.0 % of students explaining their reduced motivation to prepare.

Preparation is known to be one of the keys to successful (seminar) learning [29] and appears to influence students’ engagement in the educational activities. In seminars, engagement involves active participation in terms of peer-discussion and this requires peers to be sufficiently prepared as well. Overall, students (and teachers) were quite satisfied with the perceived preparation of peers, even though students deemed peers to be better prepared when a smaller number of students was able to finish the assignments individually. This suggests that poorly prepared students rely more on the contribution of peers. In contrast, if more students were able to complete the assignment, the preparation of peers was perceived as less satisfactory. These outcomes might imply that individuals within a group have different roles during peer-discussion. Confident students who completed their homework might function as ‘*information providers*’ or ‘*assessors*’ whereas others might be listeners or questioners [30]. These roles might be explained by the two kinds of social comparison that are described in literature, in which upward comparison is indicative for students feeling superior to peers (the information providers) whereas students feeling inferior to others (listeners) show downward comparison concerns [15]. Future research could look into these potential roles and investigate if these roles are related to self-efficacy and the preparation of the individual and their learning outcomes.

### Active participation and learning outcome

In our study seminar attendance reduced substantially over time, even though students’ motivations to actively participate were rather positive. The three arguments with the highest rating for increased motivation to participate actively were all related to peers. First, peers positively influenced participation, as they encouraged students to discuss the assignments with one another and this helped students to better understand the content. Second, the drive to be able to present the correct answer to peers during plenary discussion positively influenced students’ participation. Nevertheless, students did not want to fully rely on their peers, as indicated with the third argument ‘*I wanted to write down the correct answers myself, so I don’t have to rely on the wikis*’. This might be explained by the fact that actively participating students collected the correct answers during the sessions and refused to share their hard work with free-riders, i.e. peers whom did not attend the actual seminar but are able to collect the answers from the wiki’s [31].

Reasons severely diminishing active participation were only acknowledged by a relatively small group of students suggesting that the real factor explaining students reduced participation and attendance has not yet been identified. Still, not wanting to explain everything to (unprepared) peers was considered most demotivating according to the subgroup of students who chose to not attend the last three seminars. On the other hand, it could be that seminar attendance dropped because students were studying for the end-of-course exam. However, in the final week of the course no in-class activities were scheduled to ensure sufficient preparation time for this exam. Another reasons for reduced attendance might be the Christmas holidays, which is usually between the 5th and 6th week of the course. This could also explain why more students attended the last seminar.

### Unraveling the mechanism

Our findings show that perceived preparation of peers provides students with more confidence in finding correct answers and the perception of deepening knowledge (Fig. 2). We expected that preparation time would also influence the quality of seminars, however this was not significant. A relation between participation variables (deepening of knowledge and confidence in answers) and exam scores could not be determined, indicating that other variables affecting participation are involved. One of these additional variables might be the fact that seminars stimulated students to apply their knowledge to pathophysiology cases, as indicated by students’ explanation when elucidating why they had learned from seminars. Students also explained that the approach stimulated them to prepare better, although this was not measured in their self-reported preparation time. Students also explained that comparing and discussing seminars answers with peers (in buzz groups) was perceived very useful. However, it is interesting to notice that, when considering average scores, students were most confident in finding the correct answers and perceived to deepen their knowledge most during the fifth session (Table 3), which was attended by the lowest number of students (Table 1). This finding might be explained by the fact that teacher was ‘more visible’. One teacher noticed that students were more inclined to ask questions in this particular session, whereas in later sessions, peers often answered student questions.

In future research applying knowledge to pathophysiology cases, discussing with peers and other variables such as intrinsic motivation or conscientiousness might be examined in order to further crystalize the mechanism underlying increased exam scores due to peer-discussion.

### Limitations of this study

A threat to the validity of this study is selection bias, as the number of students participating in seminar learning substantially reduced over time. Since intrinsically motivated students are more likely to actively participate in educational activities, seminar attendance will obviously relate positively with exam scores. For that reason, we corrected exam scores with previous academic achievement, since we assume that the most motivated students in this course were more likely to be the more successful students in related courses.

Secondly, this study deals with student perceptions of the quality of seminars. It is therefore important to bear in mind that their perceptions are possibly biased by their own preparedness. Similarly, it might be difficult for students to distinguish between what was learned during seminars, and what was learned after the seminars when preparing for the exams. Still, self-reported learning outcomes are considered to be adequate and appropriate measures and are frequently examined in relation to other outcome measures, such as test scores [32].

### Future directions

A practical implication of this study, more precisely of the format for seminar learning, is that there was too much repetition. Students actually revise the preparatory assignments (homework) twice during the actual seminar, and it is known that lengthy revision particularly decreases students’ motivation to prepare [8]. A suggestion for future research includes providing students with preparatory homework consisting of relatively easy assignments followed by newly presented, and cognitively more complex assignments that will be discussed in buzz group formation such that students can demonstrate the facility and mastery of content. We anticipate that this adaptation might further improve preparation for and/or active participation in seminars.

## Conclusion

In this study peer-discussion stimulated students to participate actively, resulting in improved quality perception. Consequentially, seminar attendance was positively associated with student performance on the summative exam, even when corrected for previous academic achievement. In addition, insight into students’ motives to prepare for and participate in student-centered seminars was provided.

## Abbreviations

MCQ, multiple-choice question; N, number; h, hours; SD, standard deviation

## Additional files

Additional file 1: Student questionnaire developed for active seminar learning study. (DOC 35 kb) Additional file 2: Questionnaire teacher experience, developed for active seminar learning study (translated from Dutch). (DOC 23 kb) Additional file 3: Raw data collected via questionnaires. (XLSX 72 kb)
