# Supplementary material for: Peer-instructed seminar attendance is associated with improved preparation, deeper learning and higher exam scores: a survey study
Source: BMC Med Educ. 2016 Aug 9;16:200. doi: 10.1186/s12909-016-0715-0 (PMC4979114; doi:10.1186/s12909-016-0715-0)
Supplement: Additional file 1: — Student questionnaire developed for active seminar learning study. (DOC 35 kb) [file 12909_2016_715_MOESM1_ESM.doc]

**Additional file 1**

**Student questionnaire developed for active seminar learning study**

The table shows the relevant items of the seven student questionnaires. For each item the type of question and the number of the questionnaire (Q) are indicated. Items are translated from Dutch.

| Items | Type | Q |
| --- | --- | --- |
| 1. Indicate your personal student identification number | Open answer | 1-7 |
| 2. Were you able to complete the assignments on your own? | Yes / No | 1-7 |
| 3. How many hours did you spend preparing the assignments? | Open answer | 1-7 |
| 4. Did you attend the seminar?   - If not, indicate why you choose not to attend the seminar? | Yes / No  [open answer] | 3-7 |
| 5. Please answer the following questions on a scale from 1 to 5.   - To what extent did your peers prepare the seminar assignments? - To what extent did the seminar deepen your knowledge of the topics discussed during the lecture? - To what extent are you confident that you and your peers found the correct answer? | Scale (1-5)  1= very poorly, 5= very good  1= very little, 5= very much  1= very little, 5= very much | 1-7 |
| 6. If the seminar induces you to prepare differently, what are you planning to change and why? | Open answer | 1-6 |
| 7. If the seminar induces you to behave differently during a seminar, what are you planning to change and why? | Open answer | 1-6 |
| 8. To what extent do the following reasons improve your motivation to prepare the seminar assignments?  (All items are included in manuscript) | Scale (1-5)  1= little contribution, 5 = large contribution | 7 |
| 9. To what extent do the following reasons decrease your motivation to prepare the seminar assignments?  (All items are included in manuscript) | Scale (1-5)  1= little reduction, 5 = large reduction | 7 |
| 10. To what extent do the following reasons improve your motivation to participate actively in seminar?  (All items are included in manuscript) | Scale (1-5)  1= little contribution, 5 = large contribution | 7 |
| 11. To what extent do the following reasons decrease your motivation to participate actively in seminar?  (All items are included in manuscript) | Scale (1-5)  1= little reduction, 5 = large reduction | 7 |
| 12. Please describe to what extent you have learned from attending seminars in general? (I did / did not learn from the seminars, because …) | Open answer | 7 |
| 13. Is there anything else you would like to share with the researchers concerning seminars, wiki’s, questionnaires, etc? | Open answer | 1-7 |
