# Supplementary material for: Peer-instructed seminar attendance is associated with improved preparation, deeper learning and higher exam scores: a survey study
Source: BMC Med Educ. 2016 Aug 9;16:200. doi: 10.1186/s12909-016-0715-0 (PMC4979114; doi:10.1186/s12909-016-0715-0)
Supplement: Additional file 2: — Questionnaire teacher experience, developed for active seminar learning study (translated from Dutch). (DOC 23 kb) [file 12909_2016_715_MOESM2_ESM.doc]

**Additional file 2**

**Questionnaire teacher experience, developed for active seminar learning study** (translated from Dutch).

These are the open-ended questions asked to teachers to determine their perception of the new seminar learning format.

- How did you experience the new seminar format?
- Which changes did you observe in student behavior?
- Which changes in student behavior did you expect, but were not observed?
- What are the advantages of the new seminar format compared to seminars taught in previous cohorts?
- What are the disadvantages of the new seminar format compared to seminars taught in previous cohorts?
- How could the course be improved in the future?
- Is there anything else you would like to share with the researchers concerning the course ‘Organ systems’?
